# Testing cost containment of future healthcare with maintained or improved quality—The COSTCARES project

**DOI:** 10.1002/hsr2.309

**Published:** 2021-06-06

**Authors:** Karl Swedberg, Desmond Cawley, Inger Ekman, Heather L. Rogers, Darijana Antonic, Daiga Behmane, Ida Björkman, Nicky Britten, Sandra C. Buttigieg, Vivienne Byers, Mats Börjesson, Kirsten Corazzini, Andreas Fors, Bradi Granger, Boban Joksimoski, Roman Lewandowski, Virgilijus Sakalauskas, Einav Srulovici, Jan Törnell, Sara Wallström, Axel Wolf, Helen M. Lloyd

**Affiliations:** ^1^ Centre for Person Centred Care University of Gothenburg Gothenburg Sweden; ^2^ Department of Molecular and Clinical Medicine University of Gothenburg Gothenburg Sweden; ^3^ Department of Nursing and Healthcare, Faculty of Science and Health Athlone Institute of Technology Athlone Ireland; ^4^ Institute of Health and Care Sciences University of Gothenburg Gothenburg Sweden; ^5^ Biocruces Bizkaia Health Research Institute Barakaldo Spain; ^6^ Ikerbasque Basque Foundation for Science Bilbao Spain; ^7^ Public Health Institute Banja Luka Bosnia and Herzegovina; ^8^ Institute of Public Health Riga Stradins University Riga Latvia; ^9^ College of Medicine and Health University of Exeter Medical School Exeter UK; ^10^ Department of Health Services Management, Faculty of Health Sciences University of Malta Msida Malta; ^11^ Environmental Sustainability & Health Institute Technological University Dublin Dublin Ireland; ^12^ Department of Neuroscience and Physiology University of Gothenburg Gothenburg Sweden; ^13^ Department of Food, Nutrition and Sports Science, Center for Health and Performance University of Gothenburg Gothenburg Sweden; ^14^ Duke University School of Nursing Durham North Carolina; ^15^ Duke University Center for the Study of Aging and Human Development Durham North Carolina; ^16^ Duke University Heart and Vascular Services Durham North Carolina; ^17^ Faculty of Computer Science and Engineering Skopje North Macedonia; ^18^ Management Faculty University of Social Sciences Lodz Poland; ^19^ Voivodeship Rehabilitation Hospital for Children in Ameryka Olsztynek Poland; ^20^ Vilnius University Vilnius Lithuania; ^21^ Department of Nursing University of Haifa Haifa Israel; ^22^ Faculty of Health and Human Sciences, School of Psychology University of Plymouth Plymouth United Kingdom

**Keywords:** cost containment, health economics, health policy, health promotion, health service preventive, person‐centred care, quality of care

## Abstract

**Background:**

Increasing healthcare costs need to be contained in order to maintain equality of access to care for all EU citizens. A cross‐disciplinary consortium of experts was supported by the EU FP7 research programme, to produce a roadmap on cost containment, while maintaining or improving the quality of healthcare. The roadmap comprises two drivers: person‐centred care and health promotion; five critical enablers also need to be addressed: information technology, quality measures, infrastructure, incentive systems, and contracting strategies.

**Method:**

In order to develop and test the roadmap, a COST Action project was initiated: COST−CARES, with 28 participating countries. This paper provides an overview of evidence about the effects of each of the identified enablers. Intersections between the drivers and the enablers are identified as critical for the success of future cost containment, in tandem with maintained or improved quality in healthcare. This will require further exploration through testing.

**Conclusion:**

Cost containment of future healthcare, with maintained or improved quality, needs to be addressed through a concerted approach of testing key factors. We propose a framework for test lab design based on these drivers and enablers in different European countries.

## 1 BACKGROUND

The European Council has agreed on several values and principles regarding healthcare systems that are shared across the member states. These values include universality, access to good quality care, equity, and solidarity.^1^

At that time, costs or affordability were not explicitly addressed, although these are important issues in any system whose aim is to safeguard these common values.

The Council also stated that it is essential to make European healthcare systems financially sustainable in a way that protects future healthcare. However, expenditure for health in all European Union (EU) countries between 2000 and 2009 increased from 8.0% to 10.0% of the gross domestic product (GDP), and in the “old” EU‐15 countries alone, from 8.7% to 10.6%.^2^

In order to address important challenges affecting the future of European Health Care, a project, WE CARE funded by the FP7 programme, was initiated in 2013 and was finished in 2015.

**FIGURE 1:**
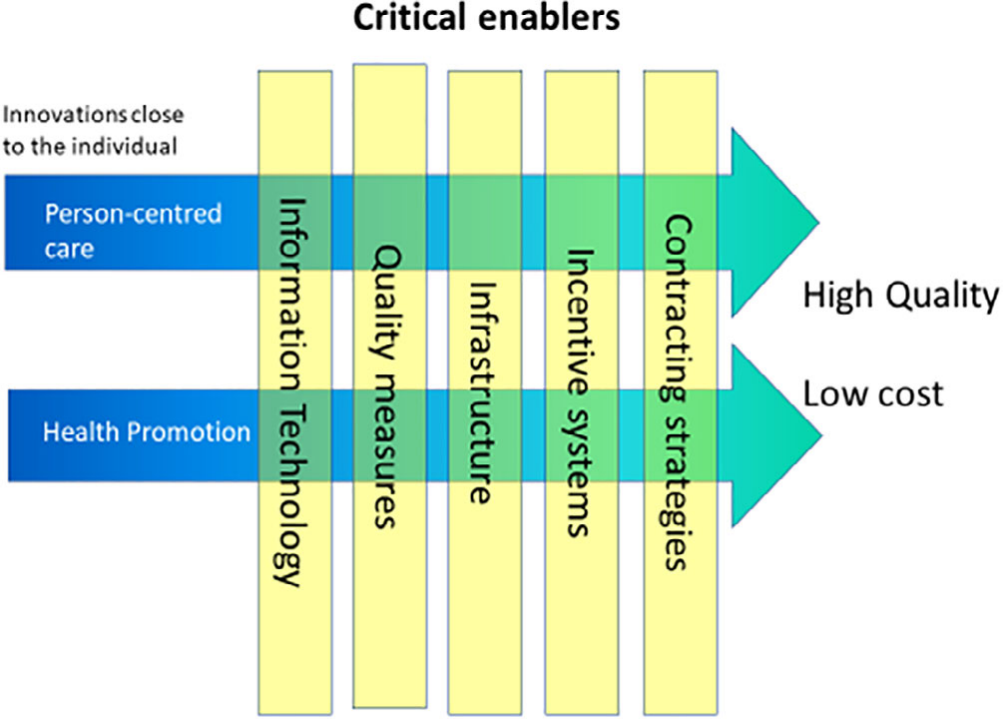
Interdependencies of macro and micro enablers and the two central innovations close to the individual (modified from WE CARE^3^, ^4^ with permission)

During the final conference in April 2015, the WE CARE consortium presented its summary report “Healthcare innovations and improvements in a financially constrained environment: Strategy Plan and R&D Roadmap”.^3^, ^4^ This report included a roadmap, which proposed a new strategic plan embedding seven interdependent themes, responsible for facilitation of a breakthrough in cost containment while, at the same time, improving the quality of care. These themes fell into two categories: (a) two drivers, which form the “backbone” of the strategic plan: person‐centred care (PCC) and health promotion and (b) five critical enablers, which are aspects of the macro environment that influence the implementation of these drivers: information technology, quality measures, infrastructure, incentive systems, and contracting strategies (Figure 1). In this paper, we explicate both PCC and health promotion, with examples, before setting out a framework for the design of test labs to put the roadmap into practice.

## 2 PERSON‐CENTRED CARE

The core component in PCC emphasizes the patient as a person in order to involve that person as a “partner.” in his/her own care and treatment. PCC is a shift away from a model, in which the patient is the passive target of a medical intervention, to an approach characterized by a “more mutual agreement,” in which the patient is an active partner in their own care and in the decision‐making process of the care and treatment plan. Co‐creation of care in the form of partnership between the patient, their family, and carer(s), and the team of health professionals caring for them, is the core component of PCC, a concept that is becoming widely used.^5^, ^6^, ^7^

PCC embodies and enacts the philosophy and ethics applied in the Capability Approach, which has been used as a theoretical frame of reference in several research disciplines, for example, in economics by the Nobel laureate Amartya Sen.^8^

PCC is the concept used in this project and is distinct from patient‐centered care, because the word “patient” tends to objectify and reduce the person to a mere recipient of medical services, or to “one who is acted on”.^6^ Today, patients often have to navigate through a fragmented healthcare system and adapt to the usual practices of healthcare organizations and professionals, rather than receiving care designed to focus on the individual patient's resources and needs, preferences, and values.^9^

The World Health Organization (WHO) uses the term “people‐centered health services” which is an approach to care that consciously adopts the perspectives of individuals, families, and communities and sees them as participants as well as beneficiaries of trusted health systems that respond to their needs and preferences in humane and holistic ways.

### 2.1 How can person‐centred care be applied?

In PCC, patients and healthcare professionals jointly develop a healthcare plan based on the patient's illness history and future goals, which identify personal resources and opportunities as well as potential barriers and needs.^5^, ^6^, ^7^

One of the fundamentals of PCC is the formation of a partnership between the patient and professionals. However, there is an asymmetry between the professional and patient. Professionals are usually in a more powerful position, as they possess greater knowledge of their specialization than the patients they serve.^10^ This implies that there cannot be a symmetrical exchange. However, a one‐way exercise of power cannot be ethically justified and will not serve either the patient or the professional. To establish a partnership requires an involvement from both parties but from different starting points and with different prerequisites. The health professional is an expert in medicine, rehabilitation, nursing, and so on, and the patient is an expert on their own life. A partnership thus demands that the patient is treated as a person, who is simultaneously capable, vulnerable, dependent, as well as independent.

In summary, PCC is operationally defined as co‐creation of care between the patients, patient proxies if appropriate, and health professionals.^6^, ^7^, ^11^

The fundamentals have been defined into three core components of PCC by Ekman et al.^6^

1. Initiating the partnership through the patient narratives.
2. Working the partnership by creating a health plan in agreement.
3. Safeguarding the partnership by documenting the health plan.

### 2.2 Effects from controlled trials

PCC represents a movement that has an explicit focus on humanizing health services and ensuring that the patient is an equal partner in their own care and treatment above and beyond care according to evidence based medicine. In this context, the body of evidence supporting the processes and outcomes associated with person‐centeredness in health and social care is constantly growing. In the cardiovascular field, PCC interventions with patients hospitalized for chronic heart failure are associated with reduced length of hospital stay, a better discharge process, and reduced patient uncertainty about their disease and treatment.^12^, ^13^, ^14^ Other outcomes include reduced healthcare costs and maintained functional performance.^15^ Furthermore, other studies involving patients with severe chronic heart failure and evaluating the core components of PCC described above found fewer hospitalizations and improved quality of life (QoL).^16^ For patients with acute coronary syndrome (ACS), a randomized controlled trial (RCT) indicated that a PCC approach was effective in increasing self‐efficacy over the whole care chain (from hospital to primary care).^17^, ^18^, ^19^ In particular, patients with lower education increased their self‐efficacy significantly more than patients with a higher level of education.^20^ A follow‐up randomized controlled trial showed lasting effects of PCC after an ACS event over the 2‐year study period.^18^

Thus, the evidence demonstrates that PCC has the potential to combine high‐quality evidence based care with controlled costs, in alignment with the aims of WE CARE and COSTCARES.

## 3 HEALTH PROMOTION

The second key driver besides PCC is health promotion. Multiple definitions for health promotion have been proposed since the term was introduced in the 1970s. One of the first definitions was given by Lalonde, the Canadian health minister in 1974 as “a strategy aimed at informing, influencing and assisting both individuals and organizations so that they will accept more responsibility and be more active in matters affecting mental and physical health”.^21^ The Ottawa Charter for Health promotion later defined Health Promotion as “the process of enabling people to increase control over, and to improve their health”.^22^

Targets for health promotion are primarily noncommunicable diseases (NCDs), which are identified as the leading causes of mortality and have several modifiable, behavioral risk factors including excessive alcohol use, physical inactivity, tobacco use, and poor diet. Biological risk factors include high blood pressure, diabetes, and obesity.^23^

Health promotion should be carried out on different levels to be effective, both population‐wide (eg, taxes, mass media campaigns, school programs) and individual, but there is uncertainty in which components are more effective. There is also a gap in research evidence from low‐ and middle‐income countries.^24^, ^25^

One very important principle of health promotion is empowerment, that is, seeking to ensure that individuals have the power to affect their own health. This aligns closely with the principles of PCC. Other important criteria include participation and having a broad perspective of health and inequality. Health promotion has gained recognition in recent years because of the growing evidence on the importance of lifestyle behavior for individual health.^26^, ^27^ In addition, socioeconomic conditions, as well as social and structural support, have been identified as important determinants of health. Thus, addressing public health in the modern era includes lifestyle behavioral changes based on a bio‐psycho‐social model.^28^

There are clear similarities between health promotion and PCC, for instance, the emphasis on identifying and supporting the individual's resources to influence their own health and the focus on the societal context affecting this process. A key component is tailoring the process to each person, exemplified by the identification of barriers and facilitators, unique to the individual, as well as the importance of the social environment for such changes to take place, for example, positive/negative reinforcement by relatives or the surrounding community.

Health promotion is included in the context of WE CARE and COSTCARES because it represents high‐quality interventions that keep populations healthy and, at the same time, means that healthcare is less costly for society. Health promotion and PCC are key drivers to cap healthcare costs, while simultaneously maintaining or improving the quality of care and resulting improved health for all.

## 4 COST ACTION 15 222 (COSTCARES)

In order to carry forward the WE CARE roadmap, Cost Action (CA) 15222 was initiated in 2017 with the project name COSTCARES. The main aim of COSTCARES is to establish processes for implementing PCC and a working framework for evaluation test labs of PCC and health promotion in different countries. These test labs are essential to the effort necessary to expand the evidence base regarding how PCC and health promotion drive cost containment in healthcare while maintaining and improving quality of care in various settings and countries. The work in COSTCARES is managed in four working groups (WGs) (See Appendix S1). The overall aim of the work of WG2 is to define a logistic and organizational framework that is necessary for the design of large‐scale testing of PCC systems that will contain costs while maintaining quality of care.

The WE CARE roadmap was developed by WG2 in reviewing the existing literature as well as practice. Examples of implementing PCC policy and practice in different settings in different countries were also identified and explored. Two successful examples/cases are outlined in Appendix S2.

## 5 FRAMEWORK FOR TEST LAB DESIGN

The test lab(s) in COSTCARES are designed to guide and stimulate the integration and collaboration between academic disciplines, industry, healthcare professionals, policy makers, and patient representatives in healthcare to achieve cost containment and quality research. COSTCARES sets out to tackle these challenges by:

1. Working toward the development of care systems based on PCC and health promotion that can be tested on a macro level.
2. Defining the parameters necessary to perform and evaluate large‐scale implementation.
3. Executing studies that will provide an adequate evidence base for PCC and health promotion across various contexts in different countries.

WE CARE posits the notion that cost containment and quality initiatives, although inextricably linked, should also be considered from a person‐centered micro level including the elements of healthcare which support preventative/health promoting strategies.^3^ It is important to consider the interdependent macro‐level enabling factors including: *information technology*, *quality measures*, *infrastructure*, *incentive systems*, and *contracting strategies* (Figure 1).

The precise design of each test lab requires a particular combination of enabling factors, underpinned by a rationale explaining how they would improve PCC and health promotion.

The hypothesized enablers in the WE CARE roadmap can be used to develop implementation strategies to overcome barriers for the effective implementation of PCC and health promotion. Just as clinical interventions are studied in randomized controlled trials, research designs exist to study the effectiveness of implementation strategies in a real‐life setting. Implementation strategies, which will likely involve one or more enablers, can be implemented sequentially, concurrently, or in an isolated fashion (depending on the programme theories to be tested). As the test lab sites will be geographically, socially, and economically disparate, the implementation strategies and role of specific enablers will differ.^29^, ^30^ What will be common to all test labs, however, is the monitoring of the core components of the PCC or health promotion intervention. Existing evidence to support the WE CARE roadmap framework for implementation of PCC and health promotion as part of the COSTCARES project is defined and discussed below.

**FIGURE 2:**
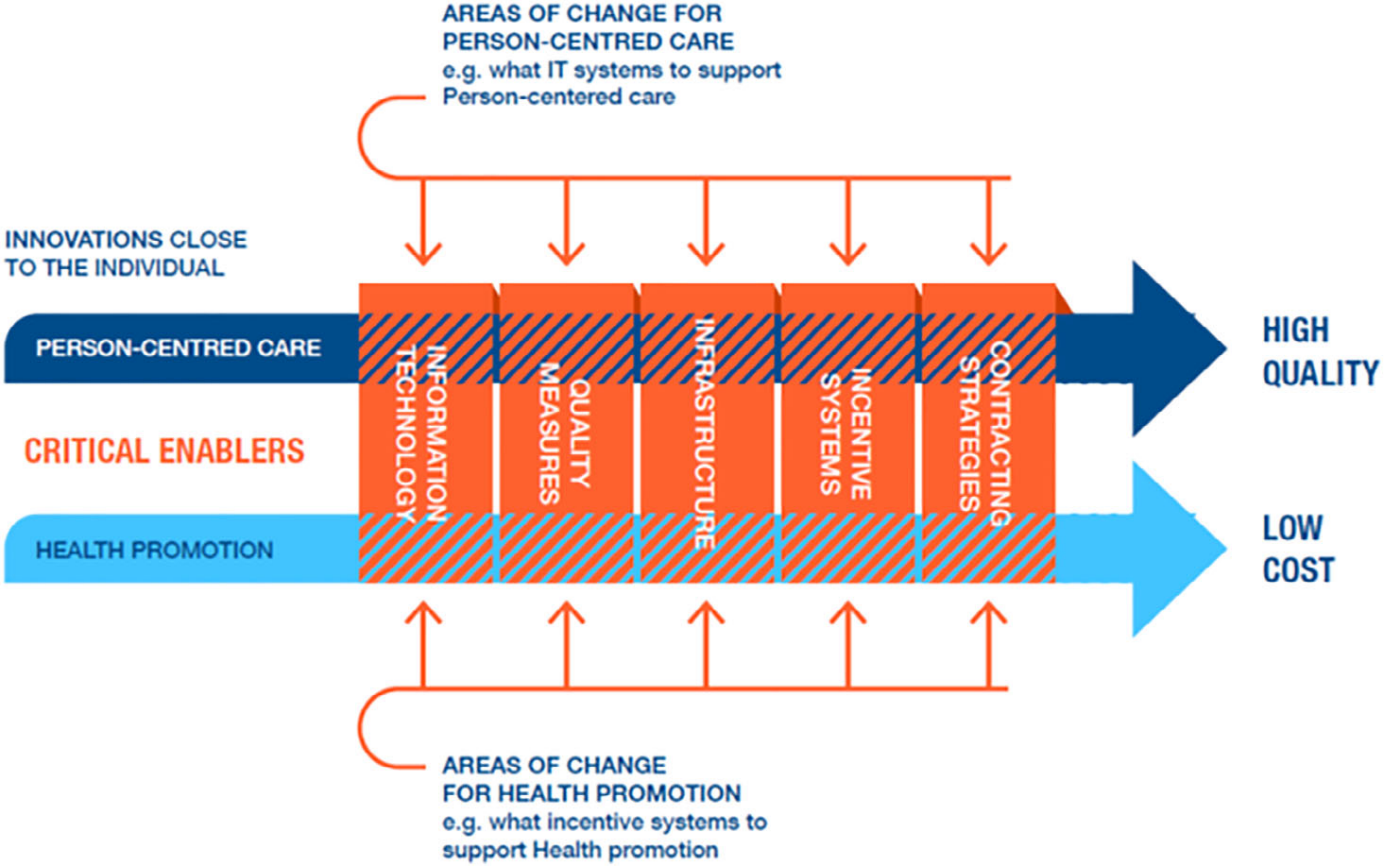
Details the critical macro enablers and the intersections with the person‐centred care and health promotion on the horizontal axis

*The macro enablers*: Each of these enablers is outlined in Figure 2 on the vertical axis and is defined below in line with current evidence and discourse. In COSTCARES, it was realized that the intersections between the enablers and the two drivers identify the core challenges in implementing the roadmap from WE CARE. These intersections are highlighted in Figure 2.

The performance in the intersections between drivers and enablers has not yet been tested. There are a number of reasons why it is difficult to develop, test, and scale‐up innovative care models. First, care systems are very complex and often highly fragmented. The model must appease the interests and diverse goals of key stakeholders underpinning the health system. Second, scientific siloes tend to result in limited interaction between vital disciplines that include medical and care services, health systems, health economics, health policy, implementation science, medical technology, information and communications technology (ICT), and communication science. Third, these care models are typically tested in smaller scale contexts with insufficient examination of the organizational, cultural, financial, technical, and legal aspects necessary to implement the model on a large scale in a real‐world setting. Thus, critical evidence to support larger scale implementation is not widely available.^31^ Innovative care models require testing on a macro level to engage policy makers, funding institutions, and care providers who can collaborate with multidisciplinary researchers to drive the systematic evaluation and practical implementation of these innovative care models. In order to develop and test such a complex intervention further, a programme theory is needed. A programme theory is an explanation, or series of linked explanations, showing how the different components of an intervention work together to produce specific outcomes. Such a model would answer the question: “How and why might this intervention (test lab) produce intended outcomes?” In addition, “What are the likely mechanisms involved?” Other relevant questions at this stage include “What existing evidence is there that this intervention might work, and can this intervention be fully described?” The latter would facilitate replication, dissemination, and implementation. These questions are answered by using a parallel process evaluation^32^ along with implementation questions that cover intervention fidelity or adaptation (was the intervention delivered as intended?), dose (how much of the intervention was delivered?), and reach (how many of the intended recipients actually received the intervention?).

*Information technology* (*IT*) encompasses a variety of technologies that include simple charting, advanced decision support, integration with medical technology, and co‐development with patients, such as mobile applications or patient‐accessible electronic health records (EHR).

The use of information technology offers great potential for reducing clinical errors (eg, prescribing errors, disease diagnostic errors), supporting healthcare professionals (eg, timely availability of up‐to‐date patient information), and collecting patient key information (symptom diaries, sensor data, digital peer‐to‐peer networks). This has increased the efficiency of care (eg, shorter patient waiting times) or even improve the quality of patient care.^33^

However, in the field of healthcare, there are also risks associated with information technology: modern information systems are costly and their failure can have a negative impact on patients and workers.^34^

The most adequate description of healthcare IT tasks is provided by the World Health Organization: the health IT is the basis for decision‐making and has four main functions^35^:

- data generation,
- compilation,
- analysis and synthesis,
- communication and use.

In addition to the integrated role of IT in clinical and diagnostic equipment, it has a unique position to capture, store, process, and timely transmit information to better coordinate health care at both the individual and population levels. For example, data mining and decision‐making capabilities can point to potential risk events for each patient, as well as contribute to the health of the population by providing insights into the causes of disease complications.^36^

Moreover, ensuring information security and privacy in the healthcare sector is becoming increasingly important. The adoption of digital patient records, tighter regulation, consolidation of providers, and the growing need for information from patients, providers, and payers point to the need for better information security. To this end, cyber security must become an integral part of patient security. Changing human behavior, technologies, and processes is part of a holistic solution.^37^

One of the most important factors in person‐centred care (PCC) and health promotion is addressing new information technology solutions enhanced by artificial intelligence (AI) to support better, safer, and more accessible healthcare.

The Information System technology vision in healthcare should highlight the changing definition of valuable care, which includes acute, chronic, and preventive care and patient health wellness promotion.^38^

*Quality measures*: In the past 5 years, many studies have been published in the area of quality measures within healthcare include the following five key dimensions aligned with COSTCARES framework: safety, equality, appropriate, person‐centred, and efficiency. Study designs are varied and include systematic reviews, cross‐sectional, prospective, and retrospective approaches with a paucity of literature regarding the methodology.^39^ Thus, future studies should consider taking into consideration specific patient safety culture measurement tools, the level of analysis, and selection of outcome measures.^40^, ^41^, ^42^, ^43^, ^44^, ^45^, ^46^ Current metrics suffer from low reliability and validity scores,^47^, ^48^ for example, the Adverse Outcome Index should be modified to more appropriately measure preventable adverse events.^49^ Moreover, health professionals, patients, and relatives should be involved in the design and collection of data^48^, ^50^, ^51^ which should include patient‐reported outcomes, morbidity, and cost,^52^ for which more recent efforts, such as the Patient‐Reported Outcomes Measurement Information System (PROMIS) measures, indicate important steps forward.^53^

*Contracting strategies*: Many healthcare systems use weighted capitation mechanisms for payment to general practitioners. In the ideal capitation model, several measures such as age, gender, morbidity, additional health needs, local labor costs, rurality, patient turnover, and so on can be included and comprehensively examined to predict patient expenditure and base capitation on the prediction.^54^ In Sweden, some argue that the current capitation function or service‐purchasing model may contribute to or increase inequality.^55^ Health economics are increasingly interested to expand evaluation of cost‐effectiveness in integrated care for chronic conditions.^56^ In the UK, the Quality and Outcomes Framework (QOF) pay for performance (P4P) scheme was explored as a potential model to reward primary care practitioners. Workers who relocate themselves on the basis of their ability may increase productivity and wages in organizations that use P4P scheme.^57^ There is a lack of knowledge about the sorting and retention effects that P4P may produce.

*Infrastructure*, *service delivery*, *and organizational models*: The fragmentation of services and providers together with shared delivery creates potential risks to the management of healthcare.^58^, ^59^, ^60^ In many national healthcare systems, the financing and operational control over different parts of the delivery of healthcare is managed by completely separate legal entities. This clearly impacts the utilization of resources. In addition, a high‐quality healthcare system requires a safe environment with sufficient technical medical equipment.^60^ From a fiscal perspective, the focus may be put on public–private partnerships, which can impact on quality, risk management, competition, and diversity. In time, this may provide service integration and an adequate welfare system (eg, support economic growth, subordinate to economic policy).^58^

*Incentive systems*: There are many types of incentive systems, typically described as financial vs nonfinancial or direct vs indirect. Good evidence regarding the effectiveness is lacking because of weak research designs. Financial incentives are most commonly applied and studied. QOF P4P showed some indication that efficient physicians may be rewarded by the system, but the study did not investigate if the overall quality increased.^57^ In addition, three Cochrane reviews concluded that there is insufficient evidence to accept or reject the use of financial incentives as a method to improve the quality of care.^61^, ^62^, ^63^ Furthermore, the cost‐effectiveness of a financial incentive model has been questioned.^62^ Regarding incentive systems for health promotion practices, Town et al^64^ conducted a systematic review of the impact of financial incentives (defined as direct payments or bonus as well as more diffuse incentives) to providers for preventive care delivery. They concluded that small rewards are likely not enough to motivate physicians to change their practice behaviors with respect to preventive care.

Furthermore, unintended consequences of introducing financial incentives into a healthcare system should be taken into account in research design. A checklist is available to determine if a financial incentive should be used and assist in its design.^65^ According to WHO Guidelines, nonfinancial incentives play an equally crucial role in incentive systems.^66^ Design of an appropriate incentive system should address to whom incentives are targeted, ongoing evaluation at multiple levels, and potential unintended consequences. It is recommended that incentives systems adhere to the four principles below^67^:

- fiscally prudent;
- simple to administer;
- culture of continuous improvement;
- equity in and access to quality care.

## 6 NEXT STEPS

COSTCARES continues to discuss the transfer and scaling up of PCC and health promotion to different contexts. Test labs will involve various alternatives to describe how the intervention and implementation of the intervention can be appropriately evaluated. In particular, COSTCARES is examining system characteristics at the micro, meso, and macro levels, including:

1. Micro—the intervention itself, for example, the types of care professionals engaged in carrying out the intervention and types of patient groups involved.
2. Meso—type of center, for example, primary care vs hospital setting.
3. Macro—country and types of healthcare policy and funding mechanisms.

## 7 CONCLUSIONS

The achievement of cost containment of future healthcare with maintained or improved quality can be addressed through a concerted approach involving several identified key factors. WE CARE identified that the fundamentals to this achievement are the drivers: PCC and health promotion. The key focus of COSTCARES is the intersections between these drivers and five critical enablers. Sustainable and efficient implementation is dependent on the interplay across these identified factors.

COSTCARES recognizes that in order to sustain the benefits of implementing PCC and health promotion, a focused approach that is cognisant of content, including geographical disparity, client care need(s), and the focus of care is necessary. In order to deliver care in a test lab scenario, it may not be feasible, or necessary, to change all enablers at once and the decision to develop implementation strategies involving certain enablers should be taken together with the stakeholders, including healthcare professionals, policy makers, and patient representatives themselves.

## FUNDING

This article/publication is based upon work from COST Action “European network for cost containment and improved quality of care,” COST15222, supported by COST (European Cooperation in Science and Technology).

GPCC is funded by the Swedish Government's grant for Strategic Research Areas, Care Sciences (Application to Swedish Research Council No. 2009‐1088) and co‐funded by the University of Gothenburg.

By the National Science Centre, Poland (Grant Number: 2015/17/B/HS4/02747) (R.L.). Duke University School of Nursing Office of Global and Community Health Initiatives (B.G. and K.C.).

N.B. was partially supported by the National Institute for Health Research (NIHR) Applied Research Collaboration South West Peninsula. The views expressed are those of the author(s) and not necessarily those of the NIHR or the Department of Health and Social Care.

The funding sources had no influence in the design, analysis, and interpretation of data as well as writing of the report or the decision to submit the report for publication.

## CONFLICT OF INTEREST

None declared.

## AUTHOR CONTRIBUTIONS

Conceptualization: Karl Swedberg, Desmond Cawley, Inger Ekman, Heather L. Rogers, Darijana Antonic, Daiga Behmane, Ida Bjorkman, Nicky Britten, Sandra C. Buttigieg, Vivienne Byers, Mats Borjesson, Kirsten Corazzini, Andreas Fors, Bradi Granger, Boban Joksimoski, Roman Lewandowski, Virgilijus Sakalauskas, Einav Scrulovici, Jan Törnell, Sara Wallström, Axel Wolf, Helen M. Lloyd

Writing—Original Draft Preparation: Karl Swedberg

Writing—Review & Editing: Desmond Cawley, Inger Ekman, Heather L. Rogers, Darijana Antonic, Daiga Behmane, Ida Bjorkman, Nicky Britten, Sandra C. Buttigieg, Vivienne Byers, Mats Borjesson, Kirsten Corazzini, Andreas Fors, Bradi Granger, Boban Joksimoski, Roman Lewandowski, Virgilijus Sakalauskas, Einav Scrulovici, Jan Törnell, Sara Wallström, Axel Wolf, Helen M. Lloyd.

  All authors have read and approved the final version of the manuscript.

  Karl Swedberg had full access to all of the data in this study and takes complete responsibility for the integrity of the data and the accuracy of the data analysis.

## TRANSPARENCY STATEMENT

The lead author affirms that this manuscript is an honest, accurate, and transparent account of the review being reported; that no important aspects of the study have been omitted; and that any discrepancies from the review as planned have been explained.

## Supporting information


**Appendix S1**: Supporting Information

## Data Availability

Data sharing is not applicable to this article as no new data were created or analyzed in this study.
