# Supplementary material for: Testing cost containment of future healthcare with maintained or improved quality—The COSTCARES project
Source: Health Sci Rep. 2021 Jun 6;4(2):e309. doi: 10.1002/hsr2.309 (PMC8180514; doi:10.1002/hsr2.309)
Supplement: Supplementary file 1 — Appendix S1: Supporting Information [file HSR2-4-e309-s001.zip › HSR2_309_210423 WG2_Framework supplementary appendix 2.docx]

## Supplementary Appendix 2

Examples of implementing PCC policy and practice

Example 1: Gothenburg, Sweden: Implementation of an intervention based on Person-Centred Care across health care levels:

The study was a two-armed randomised intervention study on three health care levels (hospital, outpatient and primary care). Eligible participants had an uncomplicated acute coronary syndrome (ACS) and were randomised to parallel groups, one control group receiving usual care and one intervention group receiving a PCC-intervention in addition to usual care (Fors et al., 2015).

The intervention was provided by staff specially prepared during a one-day introduction in the theory and practice of PCC. This was followed by four three-hour booster sessions during the study period to share experiences and maintain a continuous application of PCC. Five primary centres had designated PCC professionals [one primary care physician (PCP) and one registered nurse (RN)] who worked with the patient as a team. Five centres geographically disseminated over the Gothenburg region (population 450 000) participated voluntarily to be intervention-primary-care-centres. Patients in the intervention group participated in a PCC process emphasizing the patient as a partner through all three health care levels [hospital, outpatient and primary health care (PHC)]:

#### Hospital stay:

**Admission:** The starting point for the intervention was a structured patient narrative at admission to hospital (within 24 hours after randomisation) to *initiate the partnership*, which served as the basis for the preparation of a PCC health plan. The PCC health plan was co-created by the patient and health care professionals in order to define opportunities and barriers during recovery after ACS. The focus was on each person’s resources to achieve agreed goals during the recovery process, e.g. what activities the patient wanted to be confident enough to return to and even extend (work or leisure). The condensed narrative was compiled in the PCC health plan after the patient’s approval.

**Inpatient care:** In order to *work with the partnership,* an appointment was set between the patient, physician, and RN to consider and sign the PCC health plan, discuss the patient’s medical status, and propose a discharge date. This information was documented in the PCC health plan, which also included goals and the actions needed to accomplish them, personal resources, social network, assigned health care professionals, dates of appointments, and follow-up objectives. In addition, patients rated their symptoms, and the PCC health plan was reviewed every 48 hours and revised where necessary.

**Discharge procedure:** To *safeguard the partnership* the PCC health plan was accessible to both the patient and the health care professionals throughout the continuum of care. Medical and nursing referrals and discharge notes were shared with the patient to ensure transparency.

***Outpatient visit:***

About four weeks after discharge from the hospital, the patient met a cardiologist and a specialized RN in a team visit at the outpatient clinic. In order to *maintain the partnership,* the visit started by following up on the PCC health plan, which served as a basis for a discussion of the overall condition. If the patient´s medical status was stable, the patient was referred to the primary care setting.

Implementation at visits to primary care centre**:**

After approximately eight weeks, the patient met the specialized PCC primary care professionals at the dedicated primary care centre. To *maintain the partnership,* the goals in the PCC health plan were assessed and modified when required (e.g. divided into several minor goals to achieve them stepwise or a new goal orientation was set). The patient´s resources and support within the patient´s network and/or among health care professionals were identified to help carry out agreed upon goals. Symptoms were also reviewed. For example, if sleep disorders and/or anxiety were reported during the hospital stay, they were re-assessed and management strategies were discussed during the visit. Additional visits were scheduled if suggested by either the patient or the health care professional.

Results: A composite score of changes in self-efficacy and morbidity showed that more patients (22.3%, n=21) improved in the intervention group at 6 months compared to the control group (9.5%, n=10) (odds ratio, 2.7; 95% confidence interval: 1.2-6.2; P=0.015). The effect was driven by improved self-efficacy > 5 units in the intervention group. Overall general self-efficacy improved significantly more in the intervention group compared with the control group (P=0.026)

### Example 2: Implementation of a Health Promotion in the Basque Health Care System in Spain:

The Primary Care Research Unit of Bizkaia (PCRUB), in Bilbao, Spain, has been working to systematically study the effectiveness of a Health Promotion intervention within the local healthcare system (Ozakidetza), and specifically within primary healthcare (PHC). The team began over fifteen years ago, collecting evidence on the effectiveness of PHC strategies to enhance smoking cessation (Grandes et al., 2003) and increase physical activity (Grandes et al., 2011, Grandes et al., 2009) using clustered randomized trials. However, the primary care physicians (PCPs) who participated in the studies did not continue to utilize the Health Promotion strategies, citing lack of time, organization, communication, and/or capacity building.

In January 2006, the Basque Country Health Department commissioned the PCRUB to undertake a literature review and formative qualitative study on Health Promotion in PHC (Grandes et al., 2008). The need for mutual adaptation – to adapt an implementation strategy to the organizational structure and create organizational change to accommodate a new focus on Health Promotion in PHC was recognized. The PCRUB then began a systematic action research programme to investigate the effective integration of healthy lifestyle promotion targeting multiple risk factors into the day-to-day PHC setting – specifically smoking cessation, exercise, and healthy diet. “Prescribe Vida Saludable” (PVS) translates into Prescribing Healthy Lifestyle and involves systematic study of the effectiveness of a clinical Health Promotion intervention combined with its implementation strategy to ensure sustained uptake of the intervention.

Intervention:

The intervention is composed of multiple active measures drawn from evidence-based theoretical models and intervention strategies for health behaviour modification such as the social learning and planned behaviour theories and the 5 A’s (Ask, Advise, Agree, Assist, and Arrange follow-up) intervention framework (Goldstein et al., 1998, Whitlock et al., 2002). The intervention itself can be aligned with PCC in:

1. *Initiating the partnership.* The idea of focusing Health Promotion on primary care providers is based in the notion that the healthcare provider and patient already have an established relationship. The first “A” (ask) requires determining current levels of smoking, physical activity, and diet. For those individuals who do typically visit the healthcare centre, each participating centre determines how the partnership will be initiated. Some choose to have community agents (e.g., pharmacies, schools, parent associations, municipal sports centres) survey the individuals they have access to. Others engage the administrative assistants at the reception desk or RNs to make the first contact.

2. *Working within the partnership.* Information on current lifestyle behaviours is passed on to primary care providers in the health centre. Physicians and/or RNs (depending on the centre’s unique needs) “Advise” the individual of the risks associated with his/her current lifestyle. Working together, they “Agree” on the healthy lifestyle behaviours that require modification, if now is the right time to act, and how. The patient/person and their narratives are central to this process. If they do not feel that it is the right time to change their lifestyle, they are “advised” again at a later time and the “Agree” step is repeated as often as needed. Ownership of the lifestyle change is transferred to the person with support from healthcare professionals and the community. Agreeing on the change the individual will undertake and defining an appropriate plan and reasonable goals, consistent with the person’s needs, is a core part of the intervention. A written health plan is documented in the person’s electronic medical record by the RN or physician during the “Assist” stage.

3. *Maintaining the partnership.* The “Arrange” stage serves to maintain the partnership in a series of follow-up appointments with the RN and/or physician to review the prescribed lifestyle modification plan and its effectiveness, and determine adaptations needed to better fit the person’s needs.

**Implementation strategy**: The implementation phases that are carried out are based on the Medical Research Council’s evaluation framework (Craig et al., 2008, Moore et al., 2015). In the modelling phase, the PCPs, RN and administrative staff at four PHC centres followed an implementation strategy based on a collaborative and facilitated process, planned and designed intervention programs adapted to their specific contexts and resources, and identified strategies for change and mechanisms through which interventions should operate (Sanchez et al., 2009, Grandes et al., 2017). The RE-AIM (Reach Effectiveness Adoption Implementation Maintenance) Framework (Glasgow et al., 1999) process indicators were varied by centre, lifestyle habit, and patient characteristics.

The results of the Phase II quasi-experimental pilot trial indicated that more than half of the patients who visited a health centre (n=11,650; 51.9%) had lifestyle habits assessed; a third (33.7%; n=7,433) received advice; almost 10% (n=2,175) received a printed prescription for at least one lifestyle change (Sanchez et al., 2017). Focus groups were conducted with centre staff and 11 constructs from The Consolidated Framework for Implementation Research (CFIR) (Damschroder et al., 2009) were associated with the centre’s level of implementation performance (defined as high, medium, or low (Martinez et al., 2017). The Phase III quasi-experimental hybrid effectiveness-implementation design trial to optimize the implementation strategy has concluded in seven centres. At the time of writing, data from the health care centres offered by participating professionals and patients is being analysed.

The implementation strategy itself exemplifies the core components of PCC. The ethics that form the foundation of PCC are applied to the healthcare professionals at the local PHCs who are supported by an external facilitator to set realistic goals regarding the reach of the intervention and involve community stakeholders to develop a community of practice. Regular feedback on progress and the integration of ICT to ensure adequate data capture is also part of the implementation plan, which is developed by each PHC according to their needs. The intervention implementation process illustrates basic PCC ethics because:

1. The specific implementation strategy is decided upon by each healthcare centre according to their own characteristics. The research team supports them through the collaborative modelling process, but the centre “owns” the final implementation strategy as it is built by their team bottom up.

2. The opinions of the healthcare professionals and administrative staff are heard during the collaborative modelling discussions. The implementation strategy is adapted to their needs and environment.

3. Decision making occurs collaboratively throughout the implementation process guided by an experienced facilitator who is part of the research team. Feedback about progress on centre-defined goals of reach of the target population for each stage of the five A’s is reviewed so that the facilitator and centre implementation team can agree on action plans to improve outcomes.

Both the Gothenburg and Bilbao examples show how to implement and test PCC or a Health Promotion intervention. The emphasis in the Basque Health Care System example on the specification and study of implementation strategies adapted to the unique needs of the healthcare centres illustrates how a test lab must go beyond the study of intervention effectiveness and also examine context.

## References

CRAIG, P., DIEPPE, P., MACINTYRE, S., MICHIE, S., NAZARETH, I., PETTICREW, M. & GUIDANCE, M. R. C. 2008. Developing and evaluating complex interventions: the new Medical Research Council guidance. *BMJ (Clinical research ed.),* 337**,** a1655.

DAMSCHRODER, L. J., ARON, D. C., KEITH, R. E., KIRSH, S. R., ALEXANDER, J. A. & LOWERY, J. C. 2009. Fostering implementation of health services research findings into practice: a consolidated framework for advancing implementation science. *Implement Sci,* 4**,** 50.

FORS, A., EKMAN, I., TAFT, C., BJORKELUND, C., FRID, K., LARSSON, M. E., THORN, J., ULIN, K., WOLF, A. & SWEDBERG, K. 2015. Person-centred care after acute coronary syndrome, from hospital to primary care - A randomised controlled trial. *Int J Cardiol,* 187**,** 693-699.

GLASGOW, R. E., VOGT, T. M. & BOLES, S. M. 1999. Evaluating the public health impact of health promotion interventions: the RE-AIM framework. *American Journal of Public Health,* 89**,** 1322-1327.

GOLDSTEIN, M. G., DEPUE, J. & KAZUIRA, A. 1998. Models for provider-patient interaction: applications to health behavior change. *In:* SHUMAKER, S. A., SCHON, E. B., OCKENE, J. K. & MCBEEM, W. L. (eds.) *Models for provider-patient interaction: applications to health behavior change.* New York: Springer.

GRANDES, G., CORTADA, J. M., ARRAZOLA, A. & LAKA, J. P. 2003. Predictors of long-term outcome of a smoking cessation programme in primary care. *The British Journal of General Practice: The Journal of the Royal College of General Practitioners,* 53**,** 101-107.

GRANDES, G., SANCHEZ, A., CORTADA, J. M., BALAGUE, L., CALDERON, C., ARRAZOLA, A., VERGARA, I., MILLAN, E. & GROUP, P. V. S. 2008. Is integration of healthy lifestyle promotion into primary care feasible? Discussion and consensus sessions between clinicians and researchers. *BMC health services research,* 8**,** 213.

GRANDES, G., SANCHEZ, A., CORTADA, J. M., POMBO, H., MARTINEZ, C., BALAGUÉ, L., CORRALES, M. H., DE LA PEÑA, E., MUGICA, J., GOROSTIZA, E. & GROUP, P. 2017. Collaborative modeling of an implementation strategy: a case study to integrate health promotion in primary and community care. *BMC research notes,* 10**,** 699.

GRANDES, G., SANCHEZ, A., MONTOYA, I., ORTEGA SANCHEZ-PINILLA, R., TORCAL, J. & GROUP, P. 2011. Two-year longitudinal analysis of a cluster randomized trial of physical activity promotion by general practitioners. *PloS One,* 6**,** e18363.

GRANDES, G., SANCHEZ, A., SANCHEZ-PINILLA, R. O., TORCAL, J., MONTOYA, I., LIZARRAGA, K., SERRA, J. & GROUP, P. 2009. Effectiveness of physical activity advice and prescription by physicians in routine primary care: a cluster randomized trial. *Archives of Internal Medicine,* 169**,** 694-701.

MARTINEZ, C., BACIGALUPE, G., CORTADA, J. M., GRANDES, G., SANCHEZ, A., POMBO, H., BULLY, P. & GROUP, P. 2017. The implementation of health promotion in primary and community care: a qualitative analysis of the 'Prescribe Vida Saludable' strategy. *BMC family practice,* 18**,** 23.

MOORE, G. F., AUDREY, S., BARKER, M., BOND, L., BONELL, C., HARDEMAN, W., MOORE, L., O'CATHAIN, A., TINATI, T., WIGHT, D. & BAIRD, J. 2015. Process evaluation of complex interventions: Medical Research Council guidance. *BMJ,* 350**,** h1258.

SANCHEZ, A., GRANDES, G., CORTADA, J. M., POMBO, H., BALAGUE, L. & CALDERON, C. 2009. Modelling innovative interventions for optimising healthy lifestyle promotion in primary health care: "prescribe Vida Saludable" phase I research protocol. *BMC health services research,* 9**,** 103.

SANCHEZ, A., GRANDES, G., CORTADA, J. M., POMBO, H., MARTINEZ, C., CORRALES, M. H., DE LA PEÑA, E., MUGICA, J., GOROSTIZA, E. & GROUP, P. 2017. Feasibility of an implementation strategy for the integration of health promotion in routine primary care: a quantitative process evaluation. *BMC family practice,* 18**,** 24.

WHITLOCK, E. P., ORLEANS, C. T., PENDER, N. & ALLAN, J. 2002. Evaluating primary care behavioral counseling interventions: an evidence-based approach. *American Journal of Preventive Medicine,* 22**,** 267-284.
