# Supplementary material for: Testing cost containment of future healthcare with maintained or improved quality—The COSTCARES project
Source: Health Sci Rep. 2021 Jun 6;4(2):e309. doi: 10.1002/hsr2.309 (PMC8180514; doi:10.1002/hsr2.309)
Supplement: Supplementary file 1 — Appendix S1: Supporting Information [file HSR2-4-e309-s001.zip › HSR2_309_Supplementary Appendix 1.docx]

## Supplementary Appendix 1

### Working Groups (WG) in COST CARES and respective tasks

WG 1 : Secure funding of Labs

- 1. Establish background information for creating of a convincing package for stakeholders
  2. Create influencing toolbox (slides, elevator pitch etc)
  3. Stakeholder mapping
  4. Stakeholder interactions

WG 2: Clarify design, content and localizations of Lab

- 1. Prioritization of Intersection Points
  2. How to design a study (Lab) changing the enablers simultaneously in a large scale experimental setting?
  3. Who needs to be involved in a Lab?
  4. Where can a Lab geographically be located?

WG 3: How to assess output from labs

- 1. How to assess changes in Intersection Points (process change)
  2. How to assess cost (output of change)?
  3. How to assess all relevant aspects of quality (output change)?
  4. What national and international resources/registers can be used?

WG 4: Communication and Dissemination

- 1. Facilitate Communication internal in CostCares
  2. Facilitate communication of conclusions (ongoing and final) to stakeholders
