# Supplementary material for: Testing cost containment of future healthcare with maintained or improved quality—The COSTCARES project
Source: Health Sci Rep. 2021 Jun 6;4(2):e309. doi: 10.1002/hsr2.309 (PMC8180514; doi:10.1002/hsr2.309)
Supplement: Supplementary file 1 — Appendix S1: Supporting Information [file HSR2-4-e309-s001.zip › HSR2_309_Supplementory Appendix 3 all.pdf]

Supplementary Appendix 3

Subsequent figures/schematics in the Supplementary Appendix 3 provide hypothesized interactions between these enablers and PCC and/or health promotion. A qualitative/theory building perspective and an empirical/metrics-based quantitative evaluation can be informative in the design of test labs to examine how these contextual factors and the intervention influence one another.

It should be noted that all five enablers are not only interrelated but to some extent, they overlap with each other. This means that it is difficult to set clear boundaries between the enablers with overlap e.g. quality measures and incentives since the process of measurement itself is an incentive when it is combined with effective feedback to actors who can influence the results being tracked. Similarly, incentives and contracting strategies also overlap with each other. Although, emphasising individual enablers is important as they can indicate critical areas with the highest potential to support PCC and Health Promotion implementation.

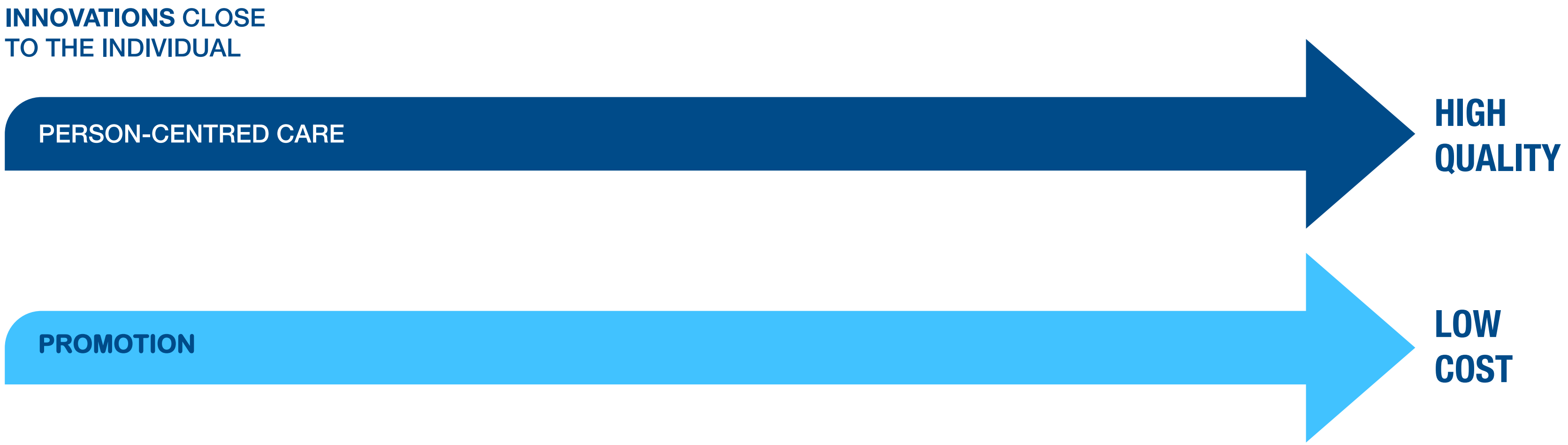
INNOVATIONS CLOSE
TO THE INDIVIDUAL
PERSON-CENTRED CARE
HIGH
QUALITY
PROMOTION
LOW
COST

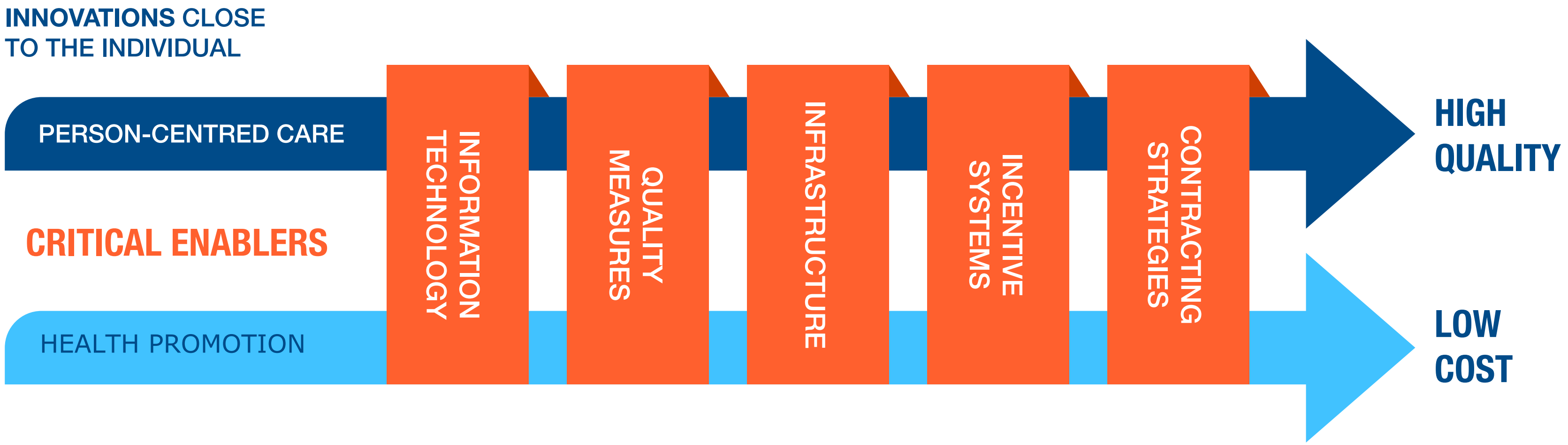
INNOVATIONS CLOSE TO THE INDIVIDUAL
PERSON-CENTRED CARE
CRITICAL ENABLERS
HEALTH PROMOTION
INFORMATION TECHNOLOGY
QUALITY MEASURES
INFRASTRUCTURE
INCENTIVE SYSTEMS
CONTRACTING STRATEGIES
HIGH QUALITY
LOW COST

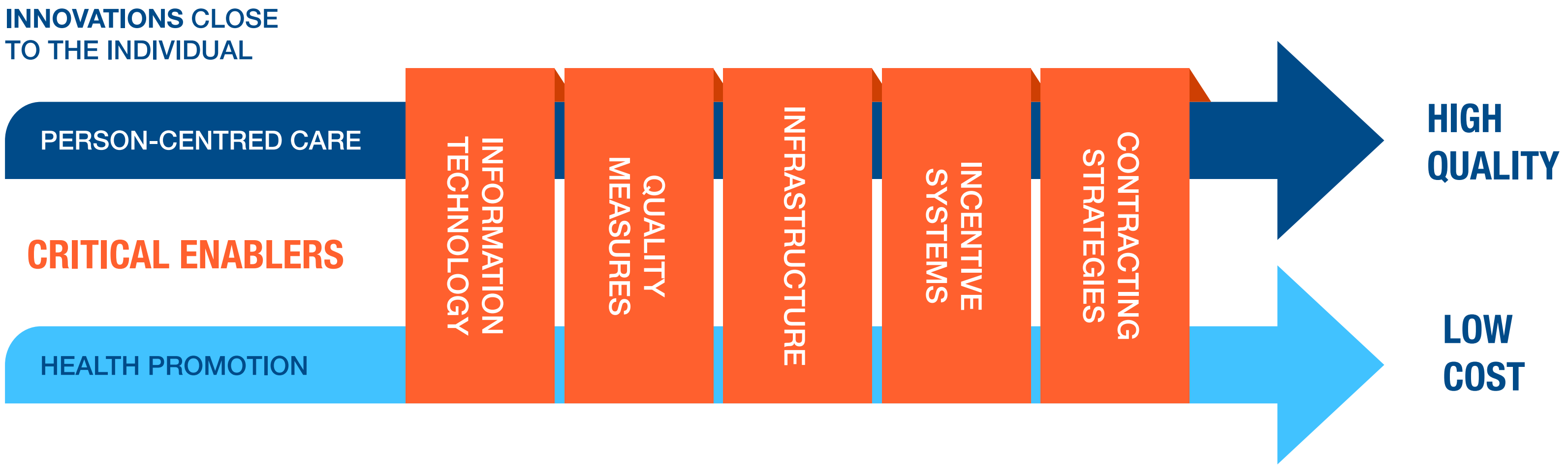
INNOVATIONS CLOSE TO THE INDIVIDUAL
PERSON-CENTRED CARE
CRITICAL ENABLERS
HEALTH PROMOTION
INFORMATION TECHNOLOGY
QUALITY MEASURES
INFRASTRUCTURE
INCENTIVE SYSTEMS
CONTRACTING STRATEGIES
HIGH QUALITY
LOW COST

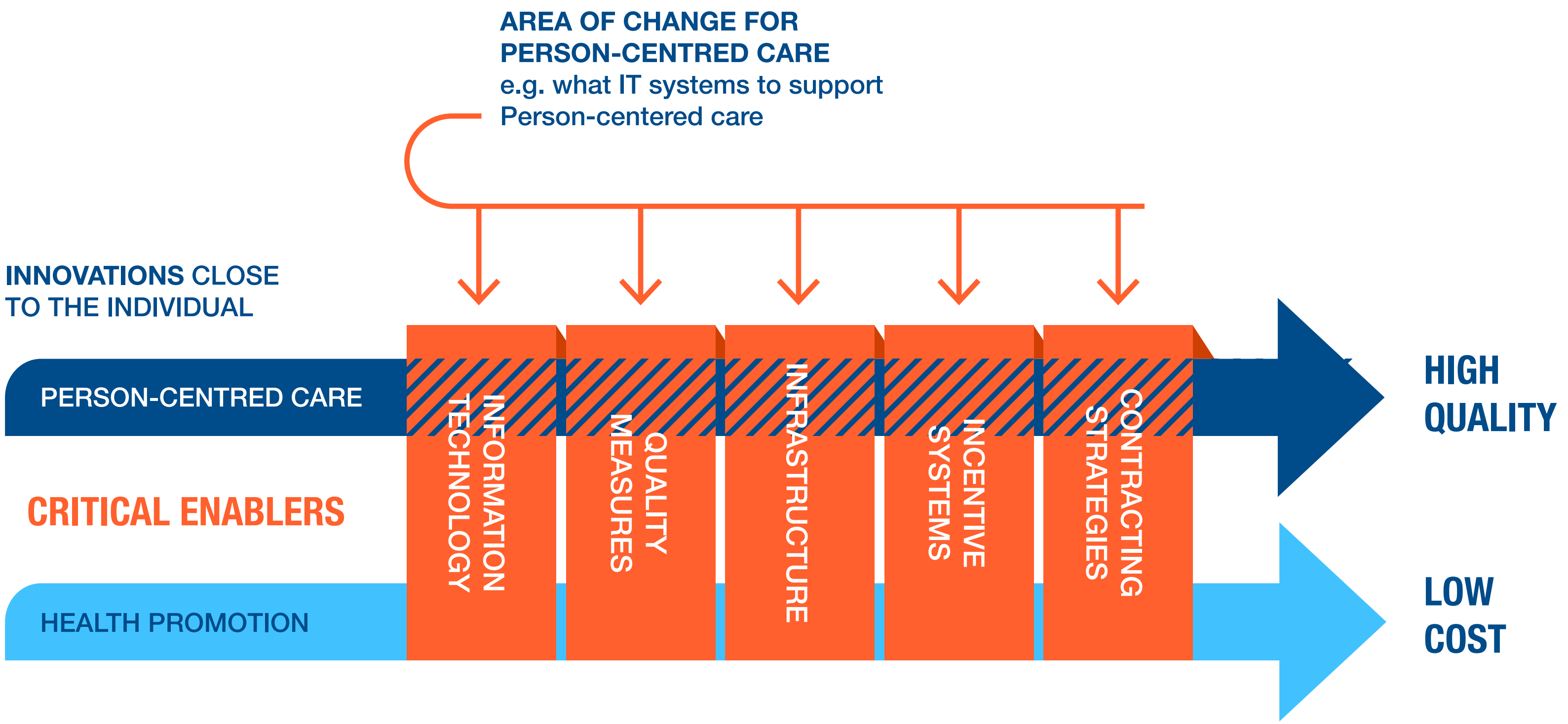

AREA OF CHANGE FOR PERSON-CENTRED CARE
e.g. what IT systems to support Person-centered care
INNOVATIONS CLOSE TO THE INDIVIDUAL
PERSON-CENTRED CARE
CRITICAL ENABLERS
HEALTH PROMOTION
INFORMATION TECHNOLOGY
QUALITY MEASURES
INFRASTRUCTURE
INCENTIVE SYSTEMS
CONTRACTING STRATEGIES
HIGH QUALITY
LOW COST

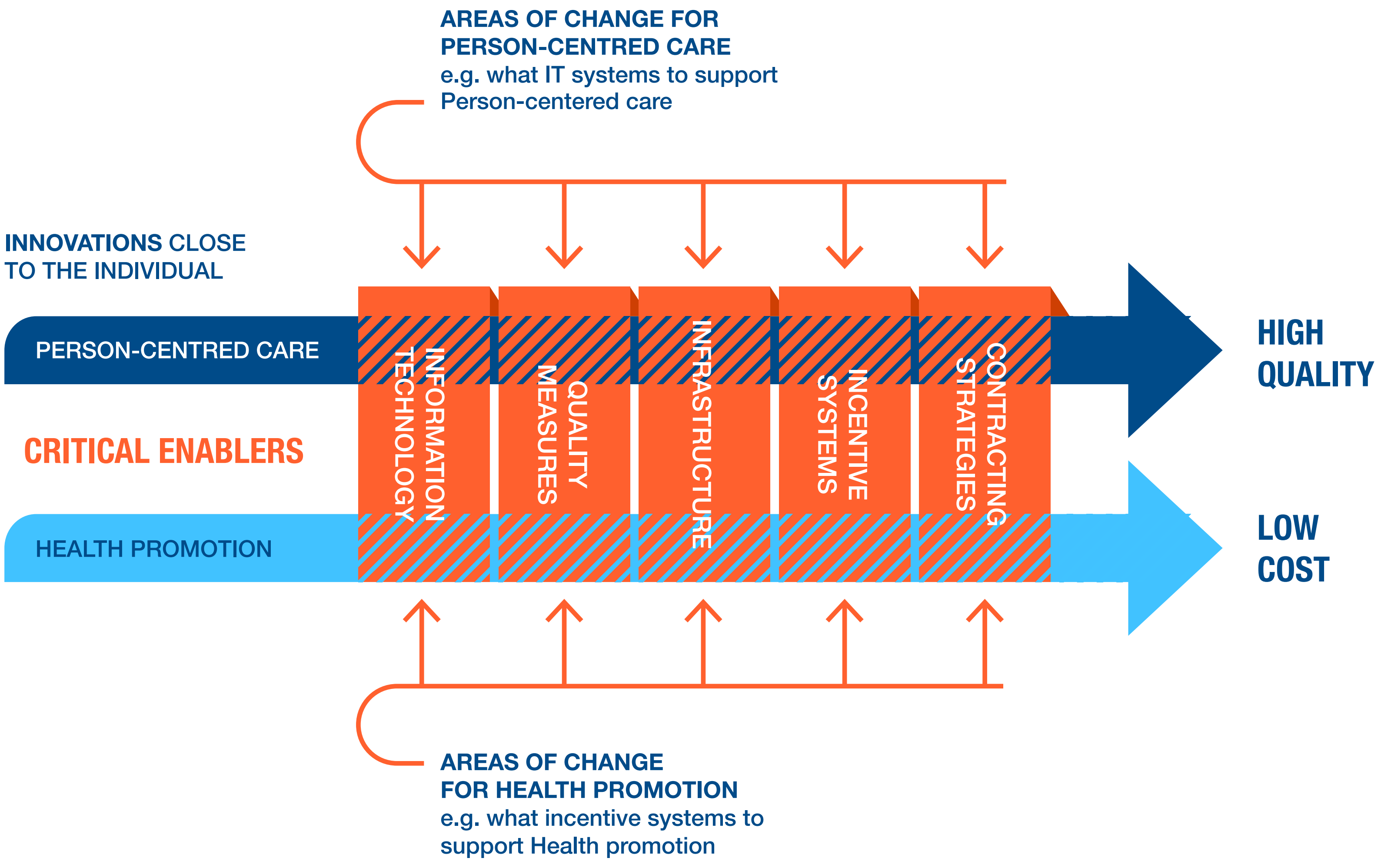

AREAS OF CHANGE FOR
PERSON-CENTRED CARE
e.g. what IT systems to support
Person-centered care
INNOVATIONS CLOSE
TO THE INDIVIDUAL
PERSON-CENTRED CARE
CRITICAL ENABLERS
HEALTH PROMOTION
INFORMATION TECHNOLOGY
QUALITY MEASURES
INFRASTRUCTURE
INCENTIVE SYSTEMS
CONTRACTING STRATEGIES
HIGH
QUALITY
LOW
COST
AREAS OF CHANGE
FOR HEALTH PROMOTION
e.g. what incentive systems to
support Health promotion

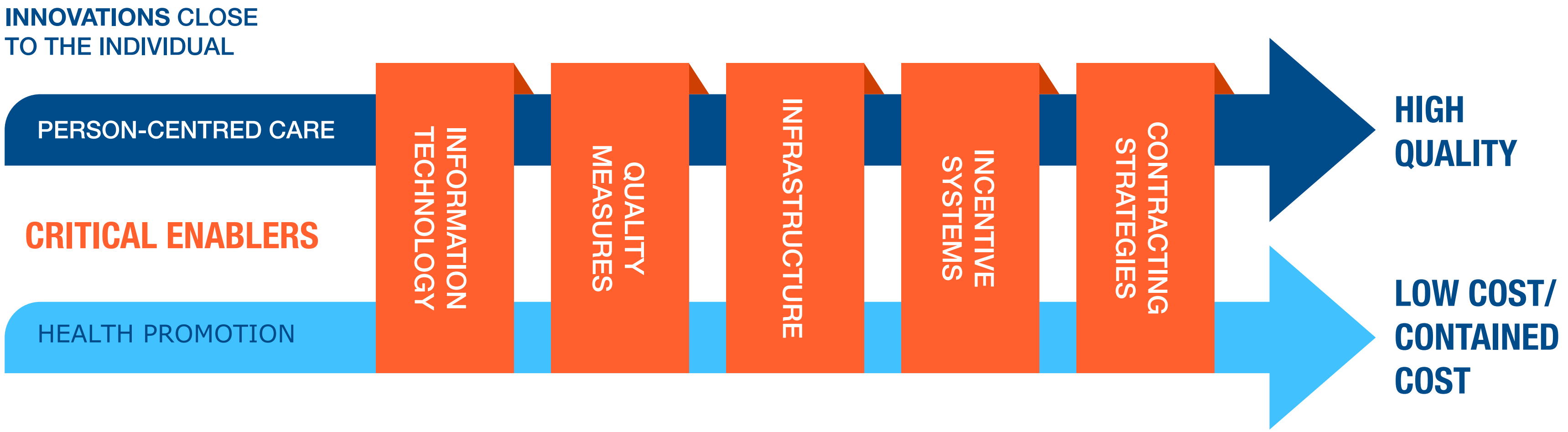

INNOVATIONS CLOSE TO THE INDIVIDUAL
PERSON-CENTRED CARE
CRITICAL ENABLERS
HEALTH PROMOTION
INFORMATION TECHNOLOGY
QUALITY MEASURES
INFRASTRUCTURE
INCENTIVE SYSTEMS
CONTRACTING STRATEGIES
HIGH QUALITY
LOW COST/ CONTAINED COST

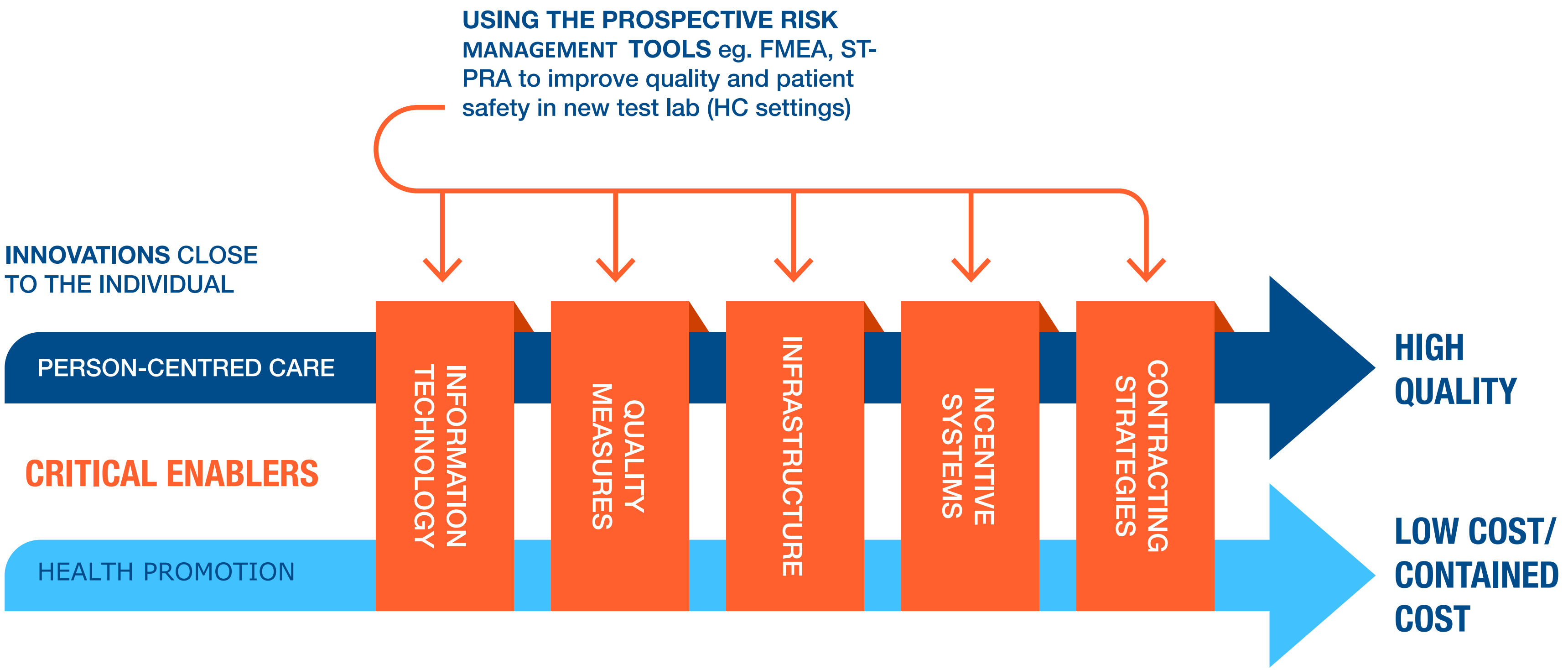
USING THE PROSPECTIVE RISK MANAGEMENT TOOLS eg. FMEA, ST-PRA to improve quality and patient safety in new test lab (HC settings)
INNOVATIONS CLOSE TO THE INDIVIDUAL
PERSON-CENTRED CARE
CRITICAL ENABLERS
HEALTH PROMOTION
INFORMATION TECHNOLOGY
QUALITY MEASURES
INFRASTRUCTURE
INCENTIVE SYSTEMS
CONTRACTING STRATEGIES
HIGH QUALITY
LOW COST/ CONTAINED COST

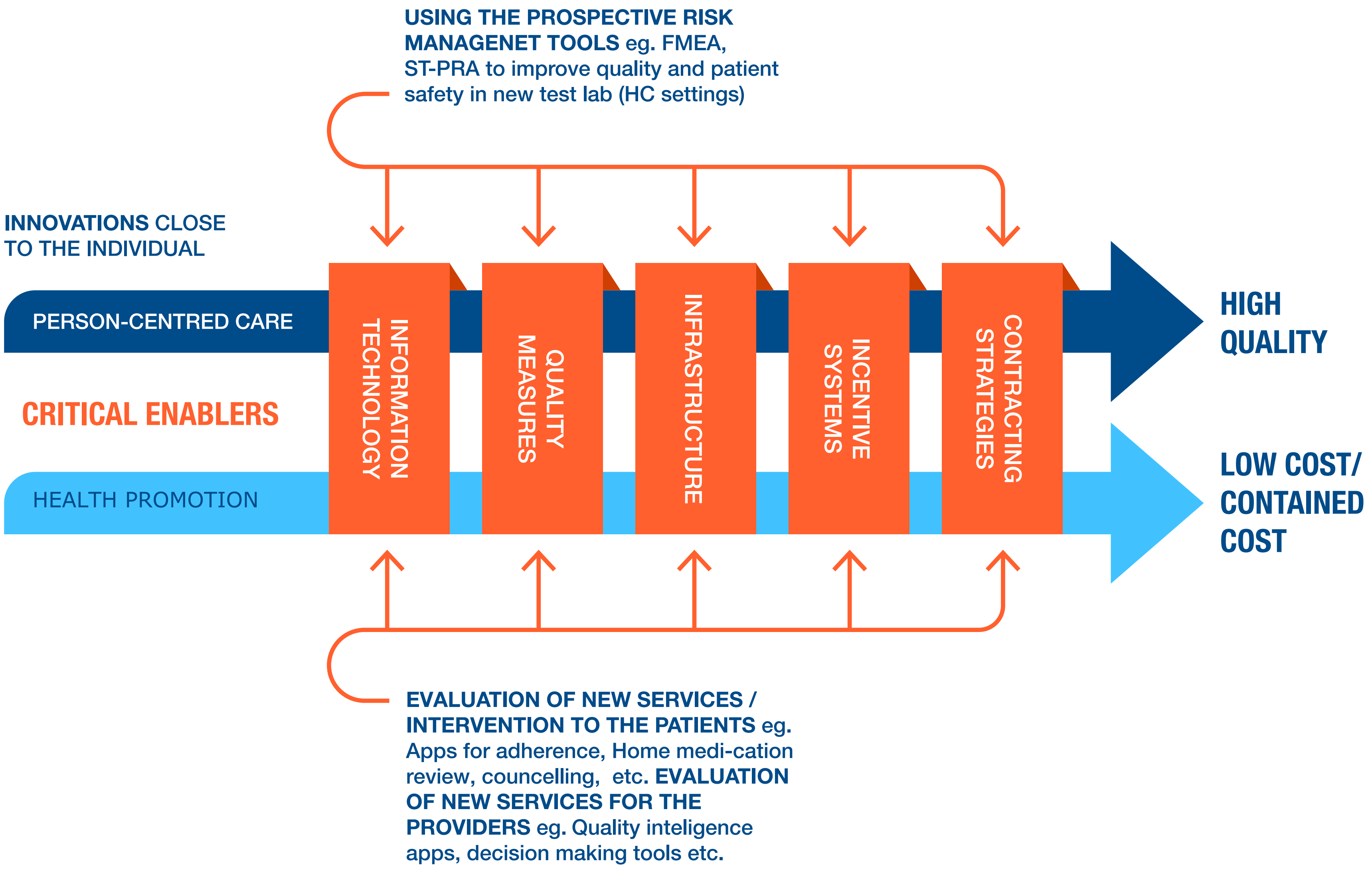
USING THE PROSPECTIVE RISK MANAGENET TOOLS eg. FMEA, ST-PRA to improve quality and patient safety in new test lab (HC settings)
INNOVATIONS CLOSE TO THE INDIVIDUAL
PERSON-CENTRED CARE
CRITICAL ENABLERS
HEALTH PROMOTION
INFORMATION TECHNOLOGY
QUALITY MEASURES
INFRASTRUCTURE
INCENTIVE SYSTEMS
CONTRACTING STRATEGIES
HIGH QUALITY
LOW COST/ CONTAINED COST
EVALUATION OF NEW SERVICES / INTERVENTION TO THE PATIENTS eg. Apps for adherence, Home medi-cation review, councelling, etc. EVALUATION OF NEW SERVICES FOR THE PROVIDERS eg. Quality inteligence apps, decision making tools etc.

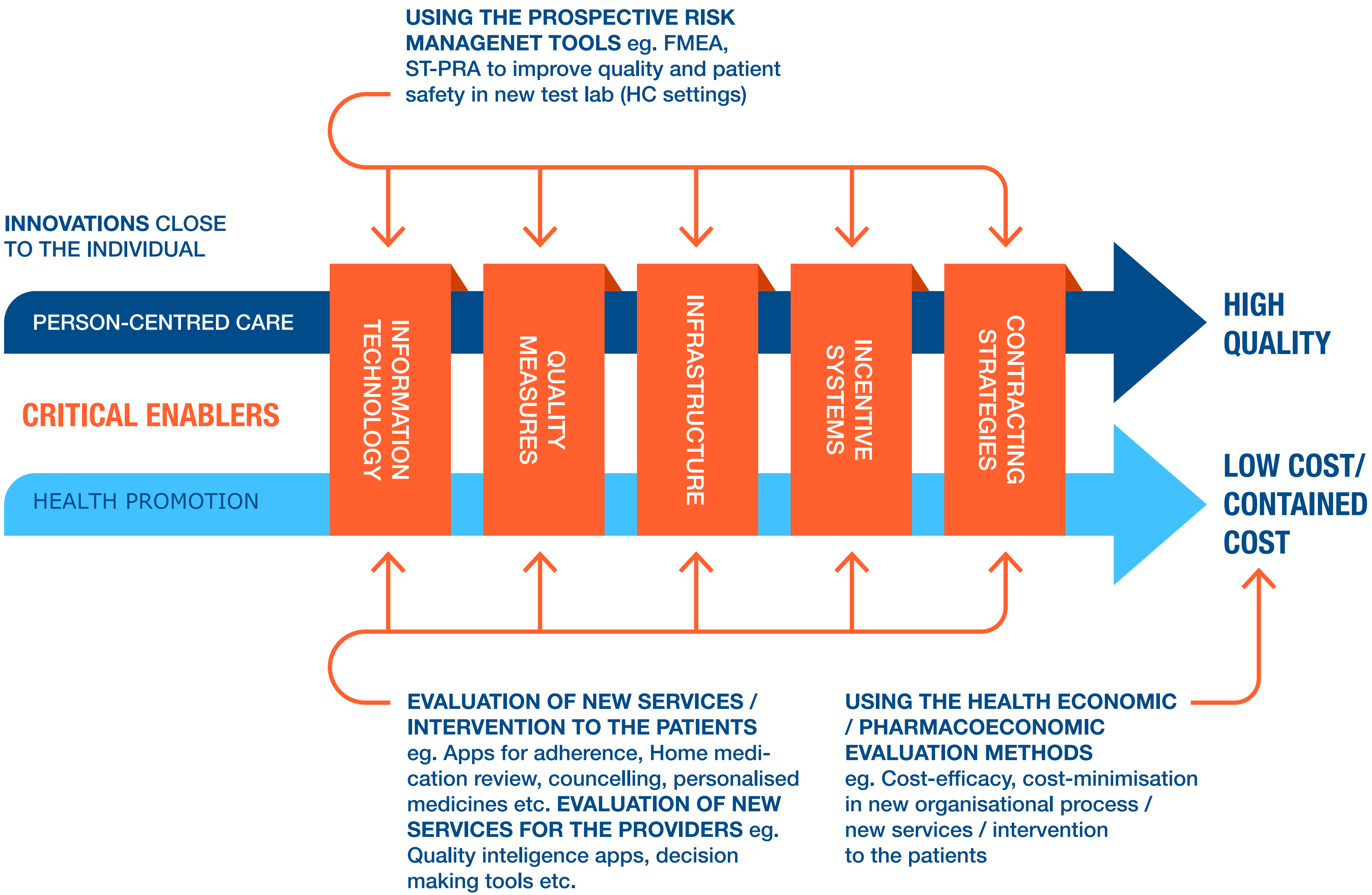
USING THE PROSPECTIVE RISK MANAGENET TOOLS eg. FMEA, ST-PRA to improve quality and patient safety in new test lab (HC settings)
INNOVATIONS CLOSE TO THE INDIVIDUAL
PERSON-CENTRED CARE
CRITICAL ENABLERS
HEALTH PROMOTION
INFORMATION TECHNOLOGY
QUALITY MEASURES
INFRASTRUCTURE
INCENTIVE SYSTEMS
CONTRACTING STRATEGIES
HIGH QUALITY
LOW COST/ CONTAINED COST
EVALUATION OF NEW SERVICES / INTERVENTION TO THE PATIENTS eg. Apps for adherence, Home medi-cation review, councelling, personalised medicines etc. EVALUATION OF NEW SERVICES FOR THE PROVIDERS eg. Quality inteligence apps, decision making tools etc.
USING THE HEALTH ECONOMIC / PHARMACOECONOMIC EVALUATION METHODS eg. Cost-efficacy, cost-minimisation in new organisational process / new services / intervention to the patients

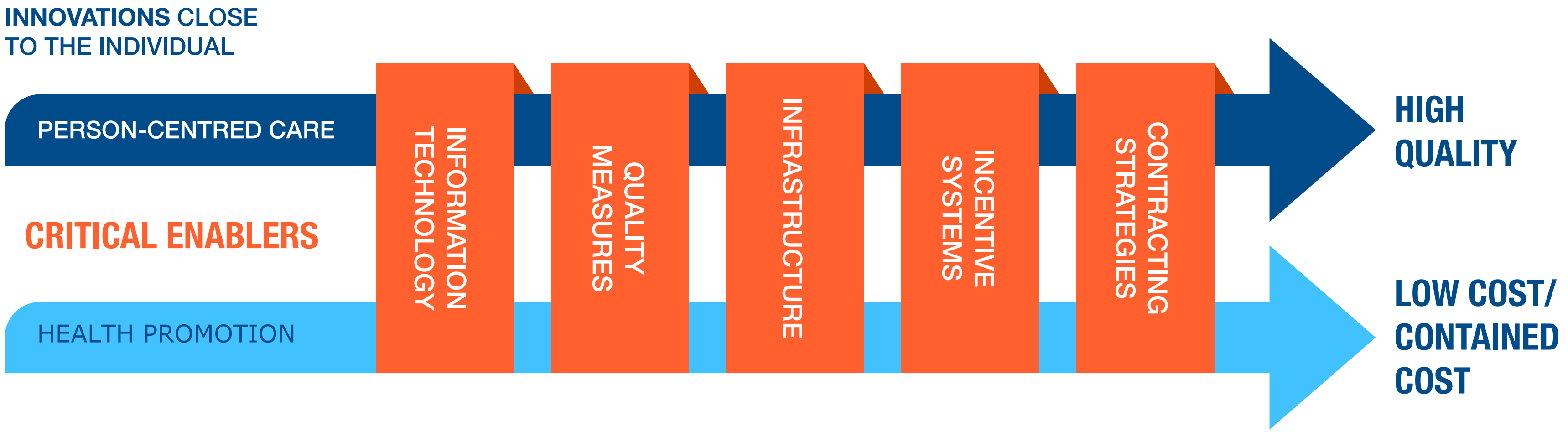
INNOVATIONS CLOSE TO THE INDIVIDUAL
PERSON-CENTRED CARE
CRITICAL ENABLERS
HEALTH PROMOTION
INFORMATION TECHNOLOGY
QUALITY MEASURES
INFRASTRUCTURE
INCENTIVE SYSTEMS
CONTRACTING STRATEGIES
HIGH QUALITY
LOW COST/ CONTAINED COST

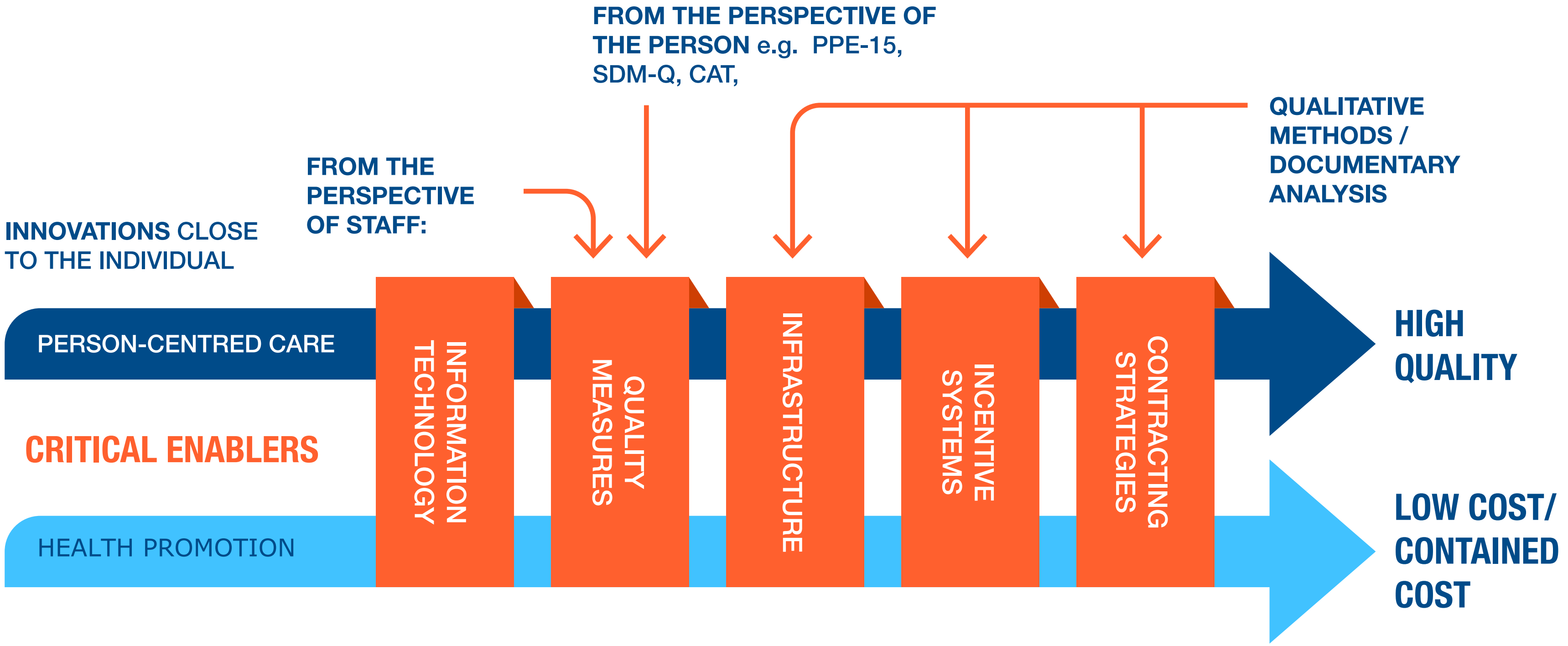
FROM THE PERSPECTIVE OF THE PERSON e.g. PPE-15, SDM-Q, CAT,
QUALITATIVE METHODS / DOCUMENTARY ANALYSIS
FROM THE PERSPECTIVE OF STAFF:
INNOVATIONS CLOSE TO THE INDIVIDUAL
PERSON-CENTRED CARE
CRITICAL ENABLERS
HEALTH PROMOTION
INFORMATION TECHNOLOGY
QUALITY MEASURES
INFRASTRUCTURE
INCENTIVE SYSTEMS
CONTRACTING STRATEGIES
HIGH QUALITY
LOW COST/ CONTAINED COST

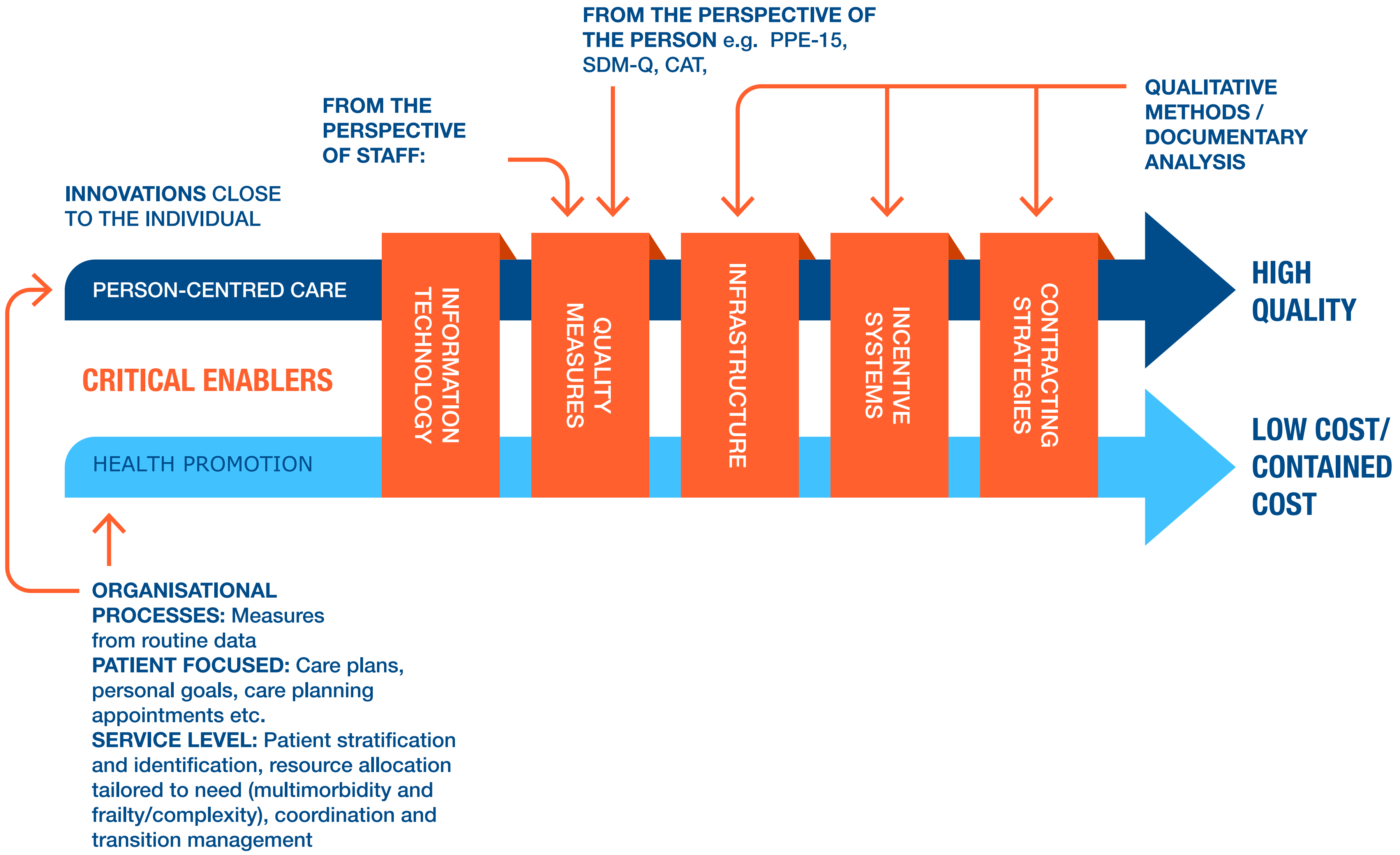
FROM THE PERSPECTIVE OF THE PERSON e.g. PPE-15, SDM-Q, CAT,
FROM THE PERSPECTIVE OF STAFF:
QUALITATIVE METHODS / DOCUMENTARY ANALYSIS
INNOVATIONS CLOSE TO THE INDIVIDUAL
PERSON-CENTRED CARE
INFORMATION TECHNOLOGY
QUALITY MEASURES
INFRASTRUCTURE
INCENTIVE SYSTEMS
CONTRACTING STRATEGIES
HIGH QUALITY
CRITICAL ENABLERS
HEALTH PROMOTION
LOW COST/ CONTAINED COST
ORGANISATIONAL PROCESSES: Measures from routine data
PATIENT FOCUSED: Care plans, personal goals, care planning appointments etc.
SERVICE LEVEL: Patient stratification and identification, resource allocation tailored to need (multimorbidity and frailty/complexity), coordination and transition management

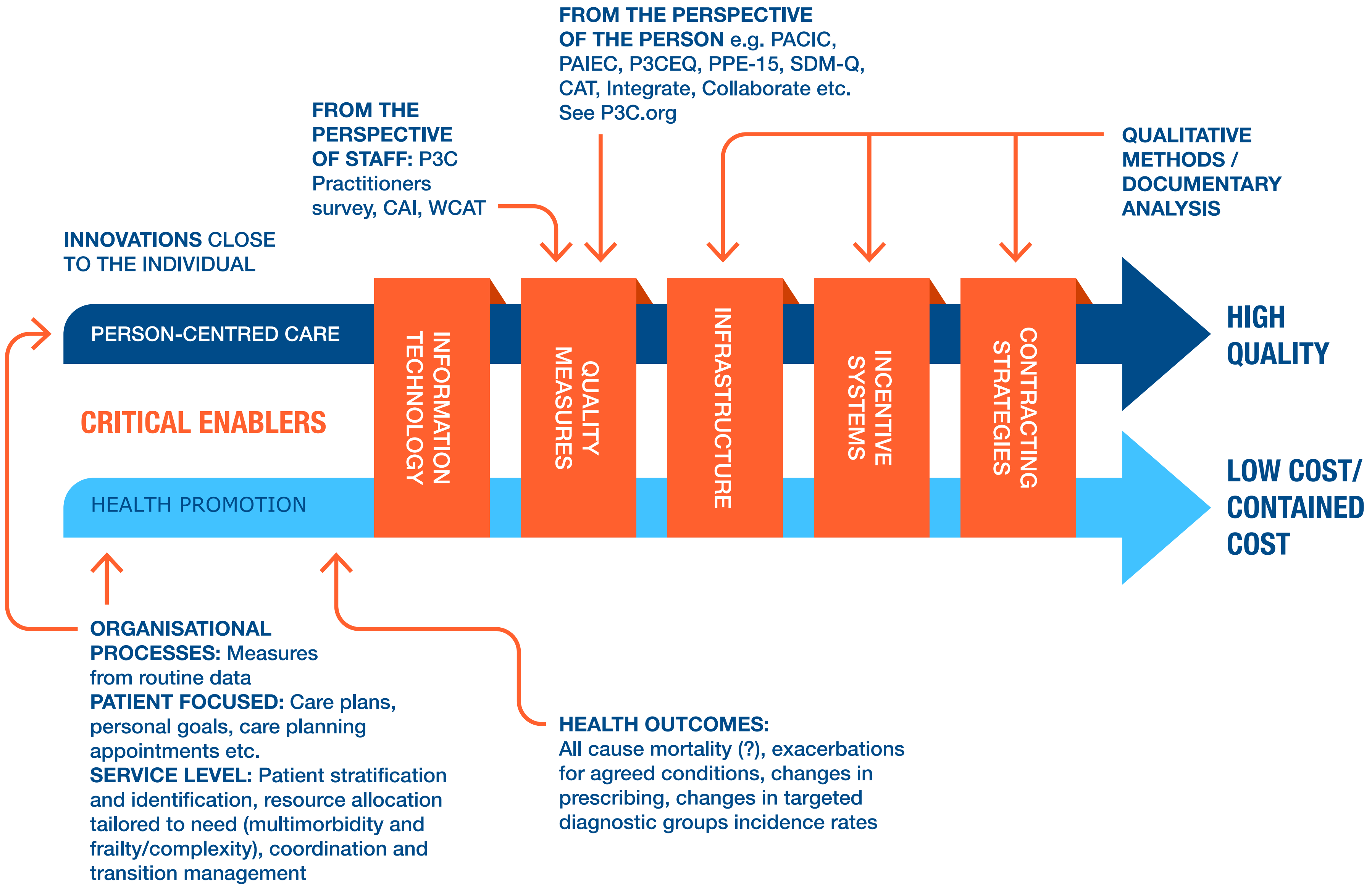
FROM THE PERSPECTIVE OF THE PERSON e.g. PACIC, PAIEC, P3CEQ, PPE-15, SDM-Q, CAT, Integrate, Collaborate etc. See P3C.org
FROM THE PERSPECTIVE OF STAFF: P3C Practitioners survey, CAI, WCAT
QUALITATIVE METHODS / DOCUMENTARY ANALYSIS
INNOVATIONS CLOSE TO THE INDIVIDUAL
PERSON-CENTRED CARE
CRITICAL ENABLERS
HEALTH PROMOTION
INFORMATION TECHNOLOGY
QUALITY MEASURES
INFRASTRUCTURE
INCENTIVE SYSTEMS
CONTRACTING STRATEGIES
HIGH QUALITY
LOW COST/ CONTAINED COST
ORGANISATIONAL PROCESSES: Measures from routine data
PATIENT FOCUSED: Care plans, personal goals, care planning appointments etc.
SERVICE LEVEL: Patient stratification and identification, resource allocation tailored to need (multimorbidity and frailty/complexity), coordination and transition management
HEALTH OUTCOMES: All cause mortality (?), exacerbations for agreed conditions, changes in prescribing, changes in targeted diagnostic groups incidence rates

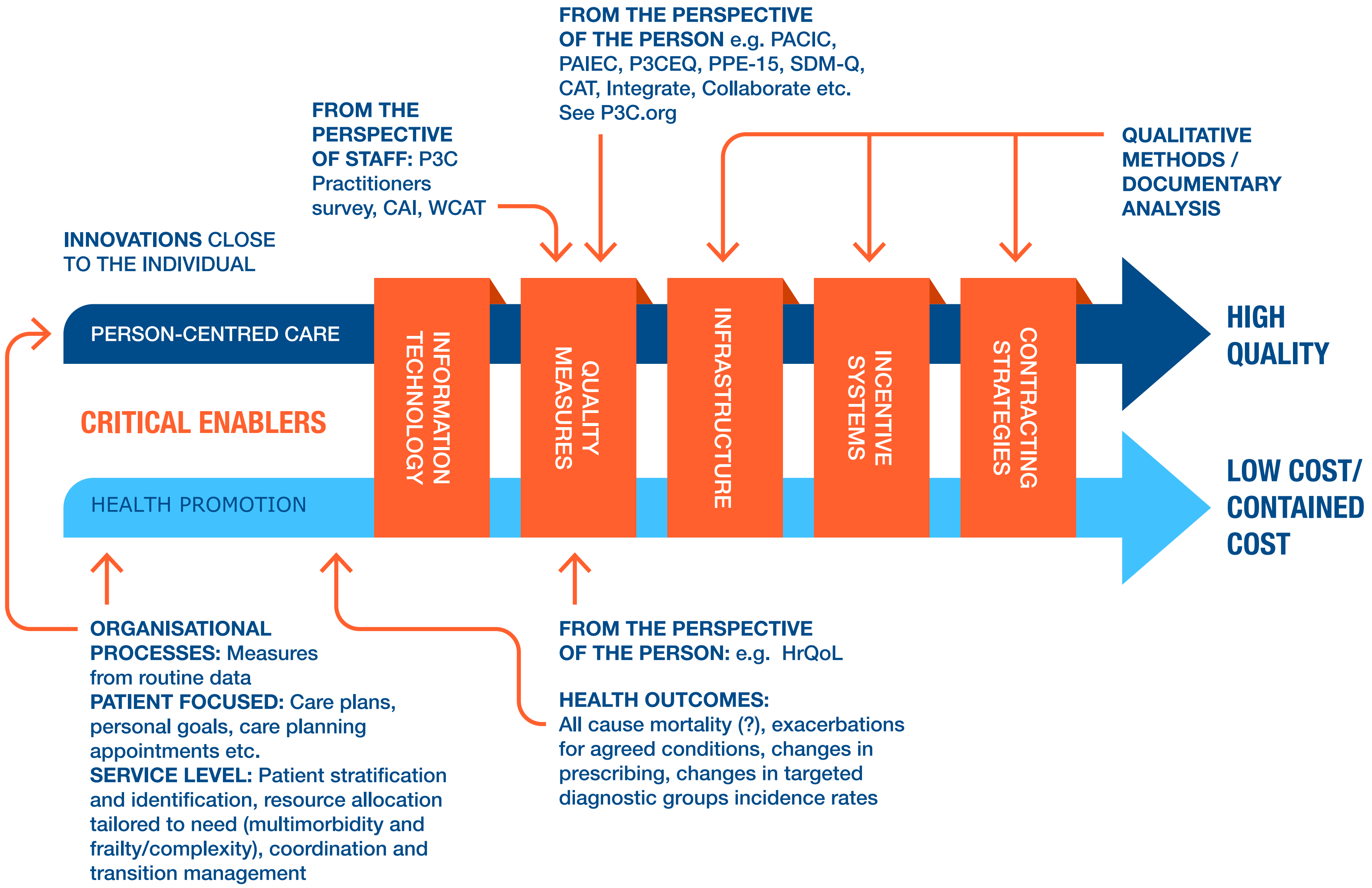
FROM THE PERSPECTIVE OF THE PERSON e.g. PACIC, PAIEC, P3CEQ, PPE-15, SDM-Q, CAT, Integrate, Collaborate etc. See P3C.org
FROM THE PERSPECTIVE OF STAFF: P3C Practitioners survey, CAI, WCAT
QUALITATIVE METHODS / DOCUMENTARY ANALYSIS
INNOVATIONS CLOSE TO THE INDIVIDUAL
PERSON-CENTRED CARE
CRITICAL ENABLERS
HEALTH PROMOTION
INFORMATION TECHNOLOGY
QUALITY MEASURES
INFRASTRUCTURE
INCENTIVE SYSTEMS
CONTRACTING STRATEGIES
HIGH QUALITY
LOW COST/ CONTAINED COST
ORGANISATIONAL PROCESSES: Measures from routine data
PATIENT FOCUSED: Care plans, personal goals, care planning appointments etc.
SERVICE LEVEL: Patient stratification and identification, resource allocation tailored to need (multimorbidity and frailty/complexity), coordination and transition management
FROM THE PERSPECTIVE OF THE PERSON: e.g. HrQoL
HEALTH OUTCOMES: All cause mortality (?), exacerbations for agreed conditions, changes in prescribing, changes in targeted diagnostic groups incidence rates
